# interRAI home care quality indicators

**DOI:** 10.1186/1471-2318-13-127

**Published:** 2013-11-19

**Authors:** John N Morris, Brant E Fries, Dinnus Frijters, John P Hirdes, R Knight Steel

**Affiliations:** 1 Institute for Aging Research, Hebrew Senior Life, Alfred and Gilda Slifka Chair in Soc Ger Research, Boston MA, USA; 2 Institute of Gerontology, University of Michigan, Ann Arbor MI, USA; 3 Ann Arbor VA Healthcare Center, Ann Arbor USA; 4 EMGO Institute for Health and Care Research, VU University Medical Center, Amsterdam, The Netherlands; 5 Department of Health Studies and Gerontology, Knowledge Exchange Chair, University of Waterloo, Waterloo, ON, Canada; 6 Hackensack University Medical Center, Hackensack, NJ, USA

## Abstract

**Background:**

This paper describe the development of interRAI’s second-generation home care quality indicators (HC-QIs). They are derived from two of interRAI’s widely used community assessments: the Community Health Assessment and the Home Care Assessment. In this work the form in which the quality problem is specified has been refined, the covariate structure updated, and two summary scales introduced.

**Methods:**

Two data sets were used: at the client and home-care site levels. Client-level data were employed to identify HC-QI covariates. This sample consisted of 335,544 clients from Europe, Canada, and the United States. Program level analyses, where client level data were aggregated at the site level, were also based on the clients from the samples from Europe, Canada, and the United States. There were 1,654 program-based observations – 22% from Europe, 23% from the US, and 55% from Canada.

The first task was to identify potential HC-QIs, including both change and prevalence measures. Next, they were reviewed by industry representatives and members of the interRAI network. A two-step process adjustment was followed to identify the most appropriate covariance structure for each HC-QI. Finally, a factor analytic strategy was used to identify HC-QIs that cluster together and thus are candidates for summary scales.

**Results:**

The set of risk adjusted HC-QIs are multi-dimensional in scope, including measures of function, clinical complexity, social life, distress, and service use. Two factors were identified. The first includes a set of eleven measures that revolve around the absence of decline. This scale talks about functional independence and engagement. The second factor, anchored on nine functional improvement HC-QIs, referenced positively, this scale indicates a return to clinical balance.

**Conclusions:**

Twenty-three risk-adjusted, HC-QIs are described. Two new summary HC-QI scales, the “Independence Quality Scale” and the “Clinical Balance Quality Scale” are derived. In use at a site, these two scales can provide a macro view of local performance, offering a way for a home care agency to understand its performance. When scales perform less positively, the site then is able to review the HC-QI items that make up the scale, providing a roadmap for areas of greatest concern and in need of targeted interventions.

## Background

This paper describes interRAI’s second-generation home care quality indicators (HC-QIs), refining and updating measures that have been in use in several countries over the prior decade. They are derived from two widely used assessments of community-dwelling persons, the interRAI Community Health Assessment (CHA) and the interRAI Home Care Assessment (HC) [1-4]. These assessments incorporate descriptive items shown to have good to excellent reliabilities [1,5].

These second-generation HC-QIs reflect a significant upgrade of the measures developed earlier [6,7]. Although many of the same concepts are addressed, in some instances the form in which the quality problem is specified has been refined, and new measures have been added. In addition, with the availability of a significantly larger cross-national dataset, the covariate structure that risk-adjusts the indicators were updated and a new, more powerful, two-step adjustment model introduced [8]. These latter improvements are critical, as the cross-site variation in a raw quality indicator value is difficult to interpret as they tangle variations in the functionality or other characteristics of the persons served with the nature of the services offered. Thus, in the current second-generation measures, each HC-QI was reviewed against a broad array of baseline measures of cognition, physical function, clinical complications, and age, with each model also including items that reference the time between assessments. With the larger sample, we were able to introduce a significantly larger array of covariates. Once these adjustors have been applied, it is reasonable to presume that significant cross-program differences in HC-QI scores are based on service diversity.

The HC-QI development sample used here is large and international in scope, including home-care clients and agencies in a number of European countries, Canadian provinces, and American states. This permitted careful and detailed review of the form of the HC-QIs and their covariates. Given the international use of these measures, a review was possible by industry representatives in multiple nations as well as members of the cross-national interRAI network. This helps to ensure that the HC-QIs reference quality issues for which a home-care service agency might reasonably be considered accountable.

The following sections describe the derivation of the HC-QIs, including the process for moving from individual interRAI assessment items through data cleaning, aggregation, and finally adjustment. Following that, the developed HC-QIs are evaluated as reasonable estimates of functional, clinical, and social outcomes. Finally, a set of overarching scales are derived, summarizing the HC-QIs within major domains of outcome. The latter is a new addition, providing a single measure against which program quality can be tracked. It responds to the need for a single measure for use in a corporate dashboard or as the initial measure for families to consider as they evaluate the overall effectiveness of their local home care program.

### Conceptual issues in creating quality indicators

interRAI has two assessment instruments for home care – the HC and the CHA. While the HC is designed for all home care clients, the CHA splits out a set of items from the HC that are appropriate for light-care clients. Included in the CHA are items that identify those persons who are in need of further assessment (a “Functional Supplement” is then triggered that will complete the HC). Thus, for a service program with a reasonable number of light-care clients, the CHA is the most appropriate assessment tool; for a heavier care population which regularly requires the full HC, the triggering mechanism for the Functional Supplement is bothersome. All the HC-QIs (and their covariates) are derived from items in the interRAI-CHA (i.e., without the Functional Supplement). Alternately stated, these HC-QIs will work for any of the many programs around the world that use either the CHA or HC assessment instruments. This was not the case for the prior generation of HC-QIs.

## Methods

### Identifying candidate HC-QIs

The first task was to identify a list of potential HC-QIs, including both change (or incidence) measures and prevalence measures. The former are based on improvement or decline between a baseline and a follow-up assessment, while the latter are based on the proportion of persons at follow-up who have a problem. In arriving at the final set of HC-QIs we began with those in the prior HC-QI generation and eventually expanded the list to include sixty-four candidate measures. Many of these measures represented alternative formulations of the same underlying concepts. The operational forms considered were all drawn from lists of quality indicators that interRAI has considered for home care, post-acute care, and long term care [6,8]. Four issues drove the decision-making process: the appropriateness of the measure in a home care environment (which ultimately causes us to exclude a number of clinical complexity and rehabilitation measures used in other settings); the breath of the coverage; the form of the measure; and the observed distribution of the candidate measures in home care. Measured that were excluded because of clinical applicability concerns included indicators that reference pressure ulcers, dehydration, delirium, and severe behavior manifestations. In terms of breath of the measures selected, the functional measures had to reference both improvement and decline, the clinical measures had to capture major life problems for persons in the community (e.g., pain, mood distress), and the social measures had to reference caregiver distress and personal isolation.

There was also a concern for the underlying problem prevalence: candidate measures with low prevalence across our multi-country data set (less than 3%) were excluded because of the scarcity of clients who demonstrated the problem. Such a measure is more akin to a sentinel event. While of interest to staff and regulators, sentinel events are poor quality indicators. With many program sites having a prevalence of zero, such indicators do a poor job in capturing the underlying latent problem. Therefore, as they arise, sentinel events are best treated on their own merit. The best quality indicators will have few sites with a score of zero and a reasonably wide variation in scores, as HC-QIs that do not vary provide no meaningful information. To assess this, in the evaluation of HC-QIs here, the scores of top performing sites were compared to the scores of the poorest performing sites; in particular, to avoid looking at the most extreme outliers, the comparison used the scores at the 5^th^ and 95^th^ position across the score distribution. Candidate HC-QIs with less than a two-fold difference in scores from the 5^th^ to 95^th^ percentile were dropped.

The next step in evaluating the candidate measures involved working with providers in a series of focus groups and one-on-one discussions to determine which of the candidate HC-QIs might be affected by their program efforts. Most of this work occurred earlier in the completion of the first generation of HC-QIs, and as there is considerable overlap in the concepts measured those earlier responses remain valid. In addition, we received more recent comments by home care providers and governmental officials using these earlier HC-QIs in several US states and Canadian provinces to help guide the current development. Here we focused on two issues: were there missing QIs that we should add and did any of the earlier QIs not work.

Finally, HC-QIs that passed these tests were reviewed by interRAI’s cross-national program development committee consisting of experts in geriatrics, gerontology, health services research, and measurement. They were asked to judge both whether each of the QIs were defined properly (e.g., the numerator worked and appropriate covariates were in place) and to judge whether the QIs would be seen by real-world practitioners and program managers to have obvious face validity when applied within the home care context. Each HC-QI had to be approved by at least 70% of the sixteen participants, including interRAI committee members from Australia, Canada, Check Republic, Finland, France, Poland, Sweden, Switzerland, and United States.

Table 1 displays the outcome areas covered by interRAI’s HC-QIs, each of which when scored represents the proportion of persons to whom the indicated condition applies. The table displays the name of the HC-QI and whether the HC-QI proportion represents a change or prevalence measure.

**Table 1:** interRAI home care quality indicators

**Home care quality indicator**	**Improvement**	**Decline**	**Follow-up prevalence**
**Functional HC-QIs**			
Instrumental activity of daily living	X	X	
Activity of daly living	X	X	
Cognition	X	X	
Communication	X	X	
**Clinical HC-QIs**			
Bladder continence	X	X	
Falls			X
Weight loss			X
Injuries			X
Mood	X	X	
Pain	X		
Daily pain, severe +			X
Pain not adequately controlled			X
**Social HC-QIs**			
Caregiver distress			X
Alone and distressed			X
Does not go out but used to			X
**Utilization HC-QIs**			
No flu vaccination			X
Hospital, emergency department, emergent care			X

### The adjustment process

If all home-care sites admitted the same client profile, in the same proportions, then adjustment would be unnecessary. Raw, unadjusted HC-QIs would suffice. Such a raw HC-QI would be no more than a simple proportion based on a numerator and a denominator. For example, a raw “Activity of Daily Living (ADL) Improvement” HC-QI would compare the number of persons who could improve in an ADL area with the number who did improve. In particular, the calculation would involve persons who had both a baseline and follow-up assessment and, in addition, who at baseline received some support with ADLs (so that they *could* improve – on interRAI assessments, this is represented by an ADL baseline score greater than zero). By way of example, suppose a given program site had 100 clients with baseline and follow-up assessment within a specified time window (e.g., six months). If 45 clients received help at baseline and 15 of these clients received no help or less help at follow-up, the numerator of the raw HC-QI would be 15 and the denominator 45. By dividing the numerator by the denominator (15/45) we have the raw ADL Improvement HC-QI proportion of .333.

But home-care sites can be expected to differ in the profile of persons served, and a critical step in the development of HC-QIs is a method to recognize differences in the baseline population – covariate adjustment. As many baseline factors may be correlated with changes in client status for each individual HC-QI, so multiple-variable covariate adjustments are needed. In earlier HC-QI work, our group employed “indirect standardization risk adjustment” using a small pool of covariates for each quality indicator, completing a simple adjustment across all cases in the site. In the second-generation HC-QIs, here the set of covariates for each HC-QI (drawing on the diverse functional, cognitive, clinical, and social items in the CHA assessment instrument) is significantly expanded and a direct stratification approach, first used by interRAI with nursing home quality indicators is employed [8]. This new approach used three direct standardization strata, differentiating by the levels of the most “disruptive” covariate (viz., the single one that is causing the largest differences) across the program sites. The covariate modeling process occurs separately within each of the three strata, generating three adjusted strata estimates for each HC-QI at any particular site. These estimates for an individual HC-QI are then pooled, weighting the contribution of the three components to match the population proportions in the three strata. This recognizes that the proportion of persons across the selected direct standardization measure for an HC-QI at any one site will diverge from the population distribution. The details of this approach are described in the following.

The process begins by selecting appropriate baseline, person-level covariates that are significantly correlated with an outcome. For example in evaluating baseline cognition we considered several different covariates: a linear summary scale; a dichotomy that emphasized low-problem persons; a dichotomy that emphasized high problem persons; and a series of neurological diagnosis dichotomies.

Candidate covariates must not be obviously correlated with the treatment approach at the home-care program sites (e.g., it would be inappropriate to use hours of physical therapy as a covariate to adjust ADL improvement). Initially, the set of covariates used in the earlier development of HC-QIs was consulted, but because of sample size limitations in the earlier work few covariates were identified. The sample size of the current study provided few such limitations and a large list of potential covariates were available for each of the HC-QIs (including the introduction of a number of summary scales and measures that asked whether the person’s served were severely impaired, almost nursing-home like in character). Also included were two measures representing the time between assessments, as different implementations of the HC and CHA around the world utilize different intervals for the assessment of clients, from three months, to six months, to one year. From this list, the initial selection of covariates was performed with a multivariate logistic equation. To be included in the provisional covariate model the baseline variables had to have a significant relationship to the HC-QI outcome. Following this, the covariates that entered the multivariate model were used to create an adjusted version of each HC-QI.

In the next step, programs with higher and lower adjusted HC-QI scores were assessed on key independent factors related to physical or cognitive function, and clinical complexity. Summary scales of those domains were used to select the single best candidate item for the direct standardization step in our process, one item for each HC-QI.

The distribution of the selected item was then used to stratify the samples into three levels – a low, mid, and high score range of the candidate direct-standardization independent variable -- and the covariate adjustment equations in each of the three strata were determined, again by multiple logistic regression. As the trichotimization can skew the distributions in as many as two of the three sub-groups, individual covariates were excluded if they had no variation within one or more strata. The covariate also was excluded if its relationship with the dependent variable was not significant in at least two of the three strata. Finally, using the three sets of estimates created by the separate covariate analysis models in each of the direct strata, and taking the population distribution of the direct standardization variable, each site’s quality indicators were weighted to a common population distribution on the direct standardization measure.

The final development effort sought to create summary HC-QI scales. Such scales permit a more global overview of a site’s quality performance, bringing together a series of related HC-QI measures. The first step in the scale construction process calls for each risk adjusted HC-QI to be normalized using a z-score transformation so that they could be directly compared. The range of each HC-QI was trichotimized so as to represent sites in the top, middle, and bottom of the z-score distribution, and scored 2, 1, and 0 respectively. In each instance, after normalization, the top score represented the most positive outcome.

Next, the trichotimized versions of the HC-QIs were subjected to a principal component factor analysis with an orthogonal varimax rotation to identify how they interrelated. Related HC-QIs were summed and the appropriateness of the scale configuration tested with the KR 20 Alpha reliability statistics. This statistic indicates the extent to which there is a consistent pattern of positive correlations among the items in the scale.

In the final analyses reported in this paper, the summary HC-QI quality scale scores are contrasted by political states/provinces pooled within wider areas (Europe, Canada, and the United States). This analysis serves as a proxy for indicating the diversity of scores to be expected in real world applications. HC-QIs can be expected to differ across countries, with countries differing in the areas in which they emphasize positive achievement.

### Data

Two data sets were used in this work, one at the client level and the second at the home-care site level. The client level data came from Europe, Canada and the US. From Europe we have two sub-sets of programs: a cross-country home care cohort (ADHOC), where each country is represented by clients from a small number of home care programs; and from Finland home care data from much of the country. In Canada, data came from all home care clients in the Provinces of Ontario and Manitoba, and a large sample of home care client sites from Nova Scotia. The home care data in the United States came from all state supported home care clients in Massachusetts, Michigan, and Georgia. These client level data were employed to identify covariates for each of the HC-QIs. This sample consisted of 335,544 home-care clients. Each of these clients had a baseline interRAI CHA (or HC) assessment and a follow-up assessment (on average about six months later). Program level analyses, where client level data were aggregated at the site level, were also based on the clients from the samples from Europe, Canada, and the United States. There were 1,654 program-based observations – 22% from Europe, 23% from the US, and 55% from Canada.

### Analysis

All of the analyses reported in this paper involve secondary data derived from interRAI’s multi-country home care data holdings. The data were provided by the sites pursuant to signed agreements with interRAI (and can only be used for de-identified research analyses), the analyses are covered by a secondary review approval, Hebrew SeniorLife Institutional Review Board and all analyses were completed using SPSS version 20.

## Results

In moving from the first to second generation HC-QIs, two types of earlier indicators were dropped. First, any measure with an underlying population, cross-site prevalence rate lower than 3% was excluded. As argued earlier such measures are more appropriate as markers of sentinel events. Among the measures so excluded where: fall requiring medical attention (2.9%); incidence of vomiting (1.1%); prevalence of physically abusing others (1.1%), prevalence of being neglected, abused, or mistreated by others (0.3%); incidence of severe malnutrition (0.6%); and incidence of burns (0.1%). Using the incidence of vomiting as an example, only 2% of the home-care sites had a score greater than zero and, of those sites with any prevalence only 17% had more than one person with the condition. Thus, the vast majority of sites had no clients with the problem and only a single person typically displayed the condition.

First generation HC-QIs also were excluded when the numerator could be triggered by more than one characteristic. For example, in the area of ADLs, two multiple-characteristic functional forms were available: (1) as an improvement indicator: the person either improved or remained independent in ADLs; or (2) as a decline indicator: the person either declined; or, failed to improve. Under either of these scenarios a site could get “credit” for something other than achieving a high ADL improvement rate or maintaining a low rate of ADL decline. It appeared better to have the two simpler forms of these measures, i.e., the person improved or the person declined. Numerically the more complex forms of the measures would have resulted in much higher rates of improvement or decline. For example, the average percent judged to improve in ADLs would be 18.7% under the recommended model vs. 58.8% for the model rejected. For decline, the rates would be either 15.6% or 40.7% respectively. On balance, we preferred that a site gets credit only when a person either became less impaired on the improvement measure or more impaired on the decline measure.

Finalizing this list of measures required both the identification of an appropriate covariate structure, the direct standardization, and, finally, testing to assure at least a two-fold difference between the best and worst performing sites for each adjusted HC-QIs.

The most appropriate baseline covariates were selected from among the broad range of CHA items. Given the large number of cases, each list could be relatively extensive ^a^. The average HC-QI has 10 covariates, with a range from 5 to 14. Covariates appearing in five or more HC-QIs (along with the number of times the covariate is employed) were as follows: one or more measures of time between baseline and follow-up (23), one or both of two age dichotomies (21), indicator of ADL status (20), clinical complexity scale (8), prior hospitalization (12), a specific IADL task (9), cognitive status (18), difficulty understanding others (6), difficulty doing cognitive IADLs (6), pain (5), ADL decline (5), unsteady gait (8), depression or sadness (15), Alzheimers or other dementia (10), and judged to be at risk of institutional placement on the Inst Risk CAP (10)^b^.

Four types of composite scales were found to work best as the direct standardization variable for trichotimization: an IADL summary score (for 8 HC-QIs), the Cognitive Performance Scale (for 8 HC-QIs) [9], a clinical risk summary scale (for 6 HC-QIs), and the ADL Long summary scale (for 2 HC-QIs) [10].

**Figure 1:**
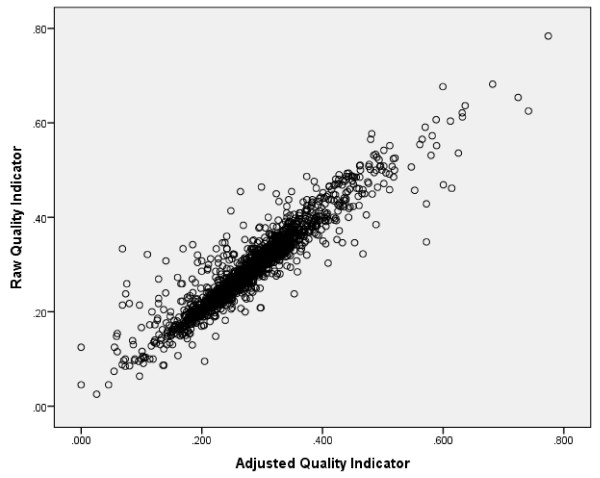
Relationship of raw and adjusted quality indicators: hospital, emergency room, and emergent care use.

Figure 1 displays a typical graph of the cross-walk between a raw and an adjusted HC-QI, here for hospital, emergency room, and emergent care (with a correlation of 0.93). For this set of twenty-three HC-QIs the mean correlation between the raw and adjusted HC-QI measures is .943 – the high is .98 and the low .91. In this application the adjustment process moves some site means up slightly, pushes others down slightly, and leaves many at about the same position.

There was a reasonably wide variation in adjusted scores for sites at either end of the problem continuum. The scores of sites at the worst end of the score range (e.g., those at the 5^th^ percentile for a given HC-QI) were compared to the scores of sites at best end of the score range (e.g., those at the 95^th^ percentile point for that same HC-QI). In all instances there was at least a two-fold difference in scores. The median score range was 5.

**Figure 2:**
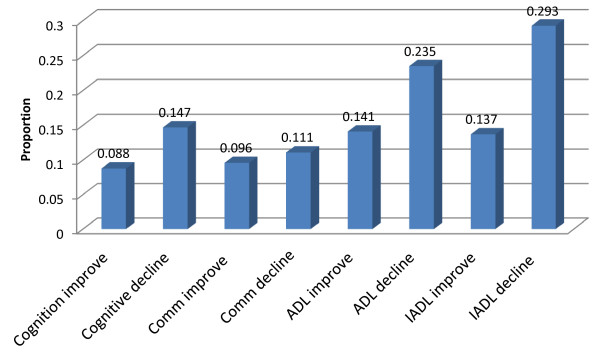
Functional quality indicators – average proportions of individuals at home care program sites declining or improving.

The next series of figures display the twenty-three HC-QIs by domain. Figure 2 displays the functional HC-QIs. Each functional HC-QI is based on a different set of cases. The four improvement HC-QIs exclude persons who were independent in the specific functional area covered by the HC-QI at baseline. The four decline HC-QIs exclude persons who were fully dependent in the specific functional area covered by the HC-QI at baseline.

When subjected to factor analysis, these two sets of HC-QIs form distinct varimax-rotated groupings – one factor references the functional decline HC-QIs, the other references the functional improvement HC-QIs. The presence of these two factors confirms that functional decline and improvement HC-QIs say something different about the performance of a home-care agency. Alternatively stated, program sites do not appear to be equally successful in improving function and preventing functional decline. The rates for all of the improvement HC-QIs and some of the decline HC-QIs tend to average around .12 (thus applying to about 12 clients out of every 100). ADL decline (a .235 prevalence) and IADL decline (a .293 prevalence) differ most from this average.

**Figure 3:**
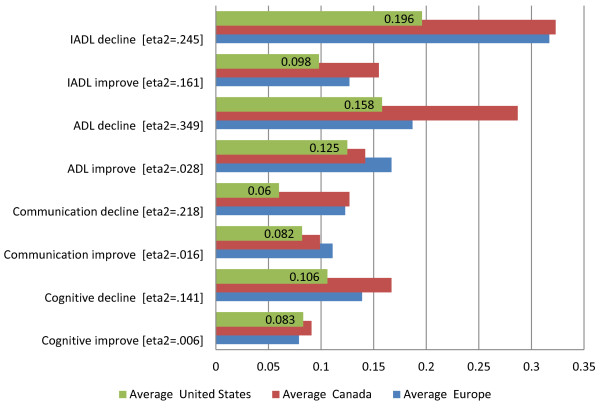
Functional quality indicators – average proportions of individuals declining or improving, by geographic region.

Figure 3 contrasts the average scores for the eight functional HC-QIs across geographic areas – representing the sites in our samples of home care programs from Europe, Canada, and the US. Here another reality emerges: all eight of the comparisons are significant, although the eta2 values -- which indicate the proportion of variance in the HC-QI score explained by the geographic area -- vary widely, from a low of .006 to a high of .349. For sites in the Canada sample, functional decline rates are higher (note the ADL Decline HC-QI has the highest eta2 value -- .349); for sites in the US sample functional decline rates are lower; and program sites in the European cohort tend to tract closer to Canada rather than US sites.

**Figure 4:**
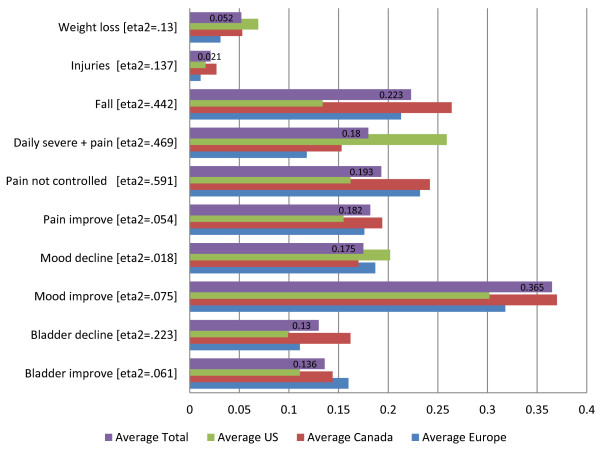
Clinical quality indicators – average proportions of individuals declining or improving, by geographic region.

**Figure 5:**
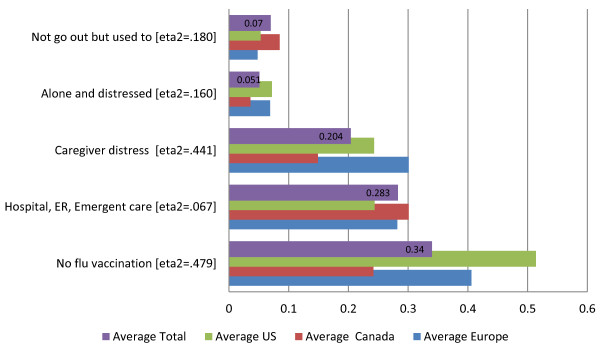
Social and service quality indicators - average proportions of individuals declining or improving, by geographic region.

Figure 4 focuses on the clinical QIs and Figure 5 on the remaining QIs, both contrasting the proportions across the same geographic regions as in the prior Figure 3. In Figure 4, the proportion of persons either with the problem or who improved in an HC-QI area ranges from about 2% for injuries and 5% for weight loss, to 13% to 20% for most of the other clinical HC-QIs, peaking at somewhat above 30% for the mood improvement HC-QI. Across geographic regions, as with the functional HC-QIs, there is a significant variation – remembering that in each instance these are combined estimates pooling for example several states in the US. The European home care program sites in our sample performed better than our sample sites in Canada and the US in the indicators of weight loss, injuries, severe daily pain, and bladder improve. US program sites in our sample had lower rates of falls and not controlled pain. Canadian program sites in our sample had a more mixed response, doing better in the areas of pain improvement, mood decline, and mood improvement, but worse in the areas of bladder decline, falls, and injuries.

Figure 5 displays the five remaining HC-QIs, relating to the social (three HC-QIs) and service (two HC-QIs) domains. Again there are significant differences in the proportion of clients with the problems and these rates differ across the geographic areas. What stands out for these HC-QIs is the substantially different proportions observed for the Canadian home care sites. On the positive side of the equation, clients at Canadian sites in our sample as compared to European and American sites in our sample are less likely to be alone and distressed (3% vs. 7% and 7%, respectively), fewer of the caregivers express distress (14% vs. 30% and 24%), and fewer clients do not receive a flu vaccine (24% vs. 40% vs. 51%). On the negative side, in Canada vs. the US more clients enter a hospital or emergency department (30% vs. 24%); and a higher proportion of persons who used to go out no longer go out of their homes (8% as compared to 5%).

The final task addressed in the analysis was the determination of summary quality scales. The detailed steps in this process are described earlier in the Methodology. Crucial to this analysis the adjusted HC-QI scores were transformed into a single unified metric by performing a z-score transformation, and each of these scores were trichotimized.

The transformed HC-QIs were next factored. The model was conditioned so that the principal component analysis would produce two varimax-rotated factors, assuming that one factor would be focused around issues correlated with preventing ADL decline and the other factor on issues that correlate with facilitating ADL improvement.

**Figure 6:**
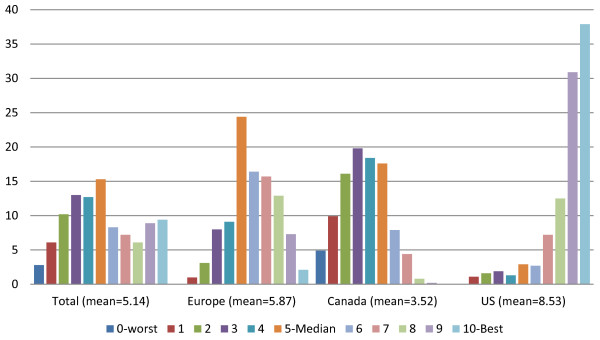
Percent sites at each level of the interRAI home care independence quality scale in total and by areas.

The varimax-rotated factor defined by functional decline (or the absence of decline actually) incorporates eleven HC-QI measures: ADL decline, IADL decline, cognitive decline, communication decline, not going out, falls, injuries, hospitalization-ED visits, mood decline, bladder decline, and pain not controlled. Referenced positively, this scale focuses on functional independence and engagement. We have labeled this scale as the interRAI Home Care Independence Quality Scale. In its raw form the scale can range from 0 to 22. In its reconfigured form it ranges from 0 to 10 (achieved by multiplying each score value by .454 and rounding the result). Figure 6 displays the distribution of this reconfigured scale for the total cohort and area samples. The scale has a mean of 5.14, a median of 5, and a superior KR 20 Alpha internal consistency reliability of .89. In line with the earlier descriptions of the HC-QIs items comprising this quality scale it is not surprising to find that programs in the US do the best, followed by programs in Europe (sig = .001, eta2 = .550). Canadian program sites in our study sample are the poorest performing in this area.

**Figure 7:**
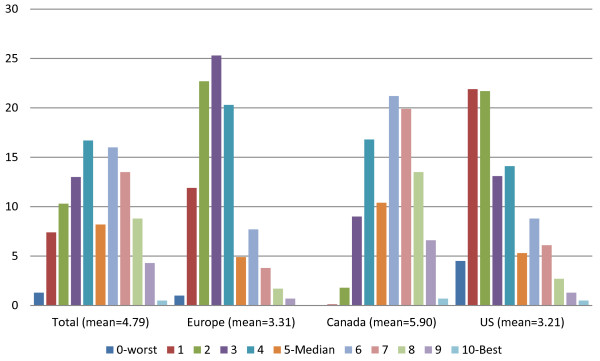
Home care clinical balance quality scale, by geographic region.

The second factor, the one anchored on the functional improvement HC-QIs incorporated nine HC-QIs – ADL improvement, IADL improvement, cognitive improvement, communication improvement, bladder improvement, mood improvement, pain improvement, caregiver not distressed, and not alone and distressed. Referenced positively, this scale indicates a return to clinical balance and it has been labeled as the interRAI Home Care Clinical Balance Quality Scale. In its raw form the scale can range from 0 to 18. In its reconfigured form it ranges from 0 to 10 (achieved by multiplying each score value by .555 and rounding the result). Figure 7 displays the reconfigured distribution for this scale for the total cohort and area samples. The scale has a mean of 4.79, a median of 6, and an acceptable KR 20 Alpha internal consistency reliability of .78. Again, in concert with the earlier descriptions of the HC-QIs items comprising this quality scale we find a reversal in findings from the prior scale. In this instance programs sites in the US and Europe perform less positively than do the programs sites in Canada (sig = .001; eta2 = .316).

Thus, the two home-care quality summary scales behave quite differently (correlation = −.59). Of the twenty-three HC-QIs in interRAI’s final set, twenty are referenced by these two scales. The three HC-QIs – weight loss, severe daily pain, no flu vaccination – are not included and thus stand on their own.

## Discussion

This paper describes a substantial improvement in interRAI’s home care quality indicators (HC-QIs). The twenty-three measures are multi-dimensional, focusing on function, clinical complexity, social life, distress, and service use. As well, the new HC-QIs have improved risk adjustment, including more complex covariate structures, longer lists of covariates (including covariates that control for varying distances between assessments), and an improved technique for adjustment.

All of the HC-QIs can be derived from two of interRAI’s most widely adopted assessment systems that have been mandated for use in countries, states and provinces across the globe. These systems are the interRAI Home Care (HC) and interRAI Community Health Assessment (CHA), with the CHA consisting of a sub-set of the HC items deemed most appropriate for the assessment of less impaired persons in the community. All of the HC-QIs can be scored using only the items in the CHA.

Significant differences were found comparing covariate-adjusted individual HC-QI outcomes for the home-care sites in our sample from Canada, Europe, and the US. By way of example, consider the HC-QIs for Canadian sites. Here, noteworthy indications of good outcomes include higher rates of pain improvement, mood improvement, and lower levels of loneliness and distress – than those in Europe and the US. However, in the similar comparison Canadian sites in our sample did poorer in a number of areas: functional decline (in IADLs, ADLs, communication, and cognition), problem rates in a number of clinical areas (bladder decline, falls, and injuries), and the proportion of persons who enter a hospital or emergency room. This type of comparison provides a window otherwise not available to a country, state, or province (or even program sites) as they seem to understand how performance compares to the performance of sites elsewhere in the world. Where outcomes are superior, one can ask why a program outshines others around the globe; and the reverse. Where outcomes are less positive, interRAI’s experience has shown that either that sites do not know they are doing well (or why), or else they expect – incorrectly – that they are doing just as well as everyone because they are comparing themselves with their own national benchmarks. The opportunity for international benchmarking with risk-adjusted quality measures opens up new opportunities to identify international best practices.

Two new summary HC-QI scales are also identified in this work, encompassing twenty of the twenty-three individual HC-QIs. The “Independence Quality Scale” is anchored on low rates of functional decline at the sites, and in addition when looked at positively also includes HC-QIs that reference going out and, low rates: for falls, injury, hospital-ED visits, mood decline, bladder decline, and pain not controlled.

The “Clinical Balance Quality Scale,” is anchored on high rates of functional improvement at the sites, and in addition to functional items incorporates the following: bladder improvement, mood improvement, pain improvement, caregiver not distressed, and not alone and distressed.

In use these two scales provide a site with a macro view of local performance, offering the first indication of how a home care agency should understand its performance. A program site could first contrast the two scale scores with their external standards (thus showing where the site fits within the larger range of scale scores) and also with the sites own performance over time. Once areas of concern are identified, the site could drill down to review the ten component HC-QIs that make up the scale, providing a roadmap for areas of greatest concern and in need of targeted interventions.

This research also provided examples of the challenges involved in using measures with low prevalence as quality indicators. In the home care environment such low-prevalence measures included: a fall requiring medical attention; physically abusing others; being neglected, abused, or mistreated by others; and vomiting. Were such measures to have been used the majority of facilities would have had a perfect score of zero and only a small number of facilities would have had more than one person with the condition. Such measures are best treated as sentinel events rather than quality indicators.

Finally, whenever HC-QIs are introduced to compare the performance of home care agencies one needs to ask to what extent an agency can be considered (or considers itself) responsible for all of the wellbeing of their clients. While functional parameters are seldom questioned, one can expect such questions to be raised on a number of the clinical and utilization HC-QIs (e.g., weight loss, injuries, not going out, or the lack of an influenza vaccination). When an agency does not consider itself accountable for one or more of these issues or is not considered for them as accountable by the insurer paying for it or the government body supervising it, one should expect to see differences in the outcome of HC-QI comparisons. The discussion very often is not taken serious since previously it could not be measured properly anyway. With the availability of the interRAI HC-QIs, this can change and the issue of accountability can be addressed and measured.

## Conclusion

For nearly all measures, the HC-QI outcomes showed significant differences at program sites (US, Europe, Canada), where the US sites in our sample generally did well at preventing decline and less well at improvement. For Canada sites in our sample the results were the reverse, and Europe was somewhere in the middle. It remains for further research to assess if existing differences in payment for home care or regulation correlate to these differences. In several European counties providing data, payment is heavily based on functional status. In these situations, there is an incentive for an earlier assessment whenever a client declines (to receive higher payments), and a disincentive for such reassessment when a client improves. This can influence the representativeness of the data. Similarly, differences in quality of the data collection can affect HC-QI results, When ‘bad outcomes’ lead to investigation or payment punishment, there will be incentives for improvement or less easily recording of decline. Whether that occurred here is unknown, but the issue needs to be considered.

Finally, no matter the challenges, the risk-adjusted HC-QIs reported here represent a significant improvement on those previously reported. With their release interRAI has established true international benchmarks.

## Endnotes

^a^The full list of covariates tested is available from the lead author on request.

^b^The list of coefficients and the associated weights are available from the lead author on request.

## Competing interests

JNM, BEF, DF, JH, and RSK are members of interRAI.

## Authors’ contributions

JNM led the study design, analysis and interpretation of data, and preparation of the manuscript. BEF, DF, JH, and RSK collaborated in interpretation of data and revision of the manuscript. The final version of the manuscript was revised and approved by all authors.

## Pre-publication history

The pre-publication history for this paper can be accessed here:

http://www.biomedcentral.com/1471-2318/13/127/prepub
